# Sources of stress in online learning scale: development and validation of an instrument to evaluate students’ stressors associated with the online learning

**DOI:** 10.3389/fpsyg.2025.1556824

**Published:** 2025-05-15

**Authors:** Ioana-Roxana Topală, Daniela-Veronica Necșoi, Ana-Maria Cazan, Maria-Magdalena Stan

**Affiliations:** ^1^Department of Psychology and Education Sciences, Transilvania University of Brașov, Brașov, Romania; ^2^Institute of Philosophy and Psychology, “Constantin Rădulescu-Motru”, Romanian Academy, Bucharest, Romania; ^3^Department of Education Sciences, The National University of Science and Technology POLITEHNICA Bucharest, Pitești University Centre, Bucharest, Romania

**Keywords:** sources of stress scale, online learning, academic stress, stressors, learning engagement

## Abstract

**Introduction:**

Since transitioning to online teaching and learning was forced to happen in the shortest ever time span during the COVID-19 pandemic, the opportunity arose for educational researchers to reflect on tools and means for measuring and explaining students’ and teachers’ relevant experiences related to the change. One of our responses to this challenge was to elaborate, administer and evaluate the psychometric properties of an instrument – Sources of Stress in Online Learning Scale (SSOLS) - that would help educational professionals assess students ‘perceptions upon sources of stress regarding their online learning. Furthermore, SSOLS’s factors would allow educators to predict students’ learning engagement due to the valid predictive association.

**Methods:**

The present research aimed to develop and test an instrument assessing the sources of stress in the context of academic online learning and to articulate its psychometric properties. The study focused on the responses of more than 500 university students regarding perceived stressors pertaining to the experience of online learning.

**Results:**

Our analysis demonstrated that the instrument (SSOLS) is reliable and valid in measuring the sources of stress associated with online learning. Also, the dimensions of SSOLS were shown to be valid predictors for academic engagement.

**Discussion:**

The significance of this study lies also in its contribution to both theoretical understanding and practical applications regarding academic stress in online learning environments.

## 1 Introduction

Online teaching and learning represented the alternative educational strategy adopted by all educational systems around the world during the COVID-19 pandemic, due to national lockdowns. The transition from physical classrooms to virtual environment was rapid and unprecedented. Some educational institutions were underprepared, the technology infrastructure was insufficient and/or inefficient, the professional readiness of teachers regarding enabling online learning was inadequate (Mustafa, 2020; UNESCO, 2020). In many cases, the suspension of face-to-face activity marked the first contact of some teachers and students with online learning and educational platforms. These are the reasons why many experts suggest that the courses and classes offered online during the crisis were not in fact online learning, but emergency remote teaching (Hodges et al., 2020). Online learning is well-planned, uses a systematic model for design and implementation and has research-backed effective results (Means et al., 2014), while emergency remote teaching is an educational alternative in a time of crises intended to maintain the continuity of instruction and to provide students the access to instruction and instructional support (Hodges et al., 2020). In short, online learning implies complex designs and a carefully decision-making process, while the emergency remote teaching is characterized by urgency, is an abrupt move to online formats that required extraordinary efforts regarding both teaching and learning. Reflecting on what distinguishes online learning from emergency remote teaching, we found that, at its core, it is a difference of purpose and, subsequently design. Online learning intends to invest in an ecosystem that supports learners yet takes time and sustained effort to identify and build (Hodges et al., 2020), relying on a systematically constructed infrastructure that pertains to the learning experience (learning content and teaching methodology, students’ interactions with the teaching staff and each other, feedback loops, other learning resources, etc.). Emergency remote teaching is a solution to address an immediate educational problem, intended not to create a robust educational ecosystem, but rather to provide temporary access to instruction and educational support, being quick to set up and reliably available during an emergency or crisis (Hodges et al., 2020). Hodges et al. (2021) underlie three essential characteristics of emergency remote teaching: its temporal nature (meaning that it is not intended as a permanent solution, only during a crisis), the immediacy of emergency (a characteristic which the long-term well planned online instruction does not have), and the remote nature of the instruction delivery (meaning that some type of technology-communications is needed to overcome the physical distance between teachers and students).

This hurried transition in the online frame challenged both teachers and students, caused an intense psychological pressure on them and raised significant concerns about their physical and emotional wellbeing and academic engagement. Although the pandemic is now over, the challenges for students, teachers and institutions providing online education remain. Online teaching and learning have emerged not just as viable alternatives in case of emergency, but as integrated components of educational systems due to their accessibility and flexibility. As online education gains steam and becomes widely used around the world, the need for accurate tools to measure relevant variables pertaining to it (student related variables, teacher related variables, institution and process related variables) becomes apparent. Instruments that target how students relate to various, specific, stressful aspects regarding learning online are scarce if not non-existent. The transition to online learning, accelerated by the COVID-19 pandemic, has underscored the importance of identifying and addressing the specific stressors that students encounter in virtual educational settings (Marinoni et al., 2020). Despite the growing body of research on student well-being, there remains a lack of reliable, context-specific instruments designed to measure these stressors comprehensively. The development and validation of the Sources of Stress in Online Learning (SSOL) scale aims to fill this gap by providing a psychometrically sound tool that captures the multifaceted nature of stress in digital learning contexts. Therefore, the present study focuses on introducing a reliable and valid instrument for measuring students’ assessment upon the stress potential of online learning. This study could contribute to both the theoretical understanding and practical application of academic stress in online learning environments by introducing a reliable tool to identify key stressors. Insights from this study could guide the development of targeted interventions to support students’ mental health and academic performance, such as enhancing teacher training programs, fostering stronger peer and instructor support networks, and creating more flexible curricula to better accommodate the demands of online learning.

## 2 Literature review

### 2.1 Online learning

Researchers agree that although consensus on a common understanding and definition of online education is purposefully sought, we are faced with its complex and dynamic nature which implies that more work needs to be done to get there (Johnson, 2019). Means et al. (2014) define online learning as the interactions the learners have with content and/or people using the Internet with the purpose of learning, as a part of formal courses or as part of the learners pursue of their interests. According to Means et al. (2014), online learning refers to both teacher-lead instruction and resources-based learning without the presence of a teacher, using the Internet. The same authors argue that the concept of online learning is similar to “web-based learning,” “cyber learning,” or “e-learning” and has a lot in common with but it distinguishes from other forms of technology-based learning like “computer-based learning,” “distance learning,” and “open educational resources.”

So, at its core, online learning is specifically designed to produce learning through digital tools in a virtual environment. Online learning considers and develops virtual learning context pertaining to educational objectives and, ideally, students ‘psychosocial specificities. Online learning, same as face-to-face learning, is the result of pedagogical craftmanship which seeks to design specific relevant learning contexts for meaningful learning experiences.

### 2.2 Online learning and blended learning approaches (pros and cons)

Based on their meta-analysis on the effectiveness of blended instruction combined with the results of other meta-analysis or individual studies on distance or online learning, Means et al. (2014) generated a model which can be used in analyzing the diversity and the effectiveness of different online learning experiences. The model comprises a set of nine instructional design dimensions which combined can give a measure of the effectiveness of different online learning settings: *modality* (fully online/ blended learning with different percentages of online or face-to-face learning), *pacing* (self or class paced learning), *student-instructor ratio*, *pedagogy* (teacher centered/student centered), *instructor role online* (from non-existent to an active one), *student role online* (passive or active role), *online communication synchrony* (synchronous, asynchronous or both), *role of online assessments* (student’s preparedness/basis for adaptive measures/ grading, etc.), and *source of feedback* (automated/ delivered by teacher or students or both). Also, these dimensions could moderate the online learning various effects. The advantages of different kinds of online learning experiences can be identified as: the ability to render concrete visual representations of abstract concepts; increased interactivity between the learner and the content to be learned; immediate feedback for students and instructors; customizability of the pace, content complexity, interface, and amount of scaffolding for individual learners; ability to immerse the learner in complex, lifelike environments and challenges; automated recording of detailed data of each learner’s interactions on the learning system over time (Means et al., 2014). In a report based on an inquiry of institutional leadership regarding the impact of digitalization on European higher education, researchers found that amongst top three effects of digitally enhanced learning and teaching were (1) encouragement of revision of teaching methods and pedagogical innovation; (2) giving teaching an enhanced flexibility, regarding time and place; and (3) enabling better monitorization of study progress and student learning (Gaebel et al., 2021).

Recent systematic research review on challenges students faced in the *online component* of blended learning, found five inter-related categories of challenges: (1) self-regulation challenges (procrastination, improper time management, improper utilization of online peer learning and online help-seeking strategies, etc.), (2) technological literacy and competency challenges (handling different user interfaces, resistance to technology, poor understanding of directions and expectations in online learning, tec.), (3) students isolation challenges (students feeling unease in using synchronous online communication tools, students feeling uncomfortable being the center of attention etc.), (4) technological sufficiency challenges, (5) technological complexity challenges (Rasheed et al., 2020). In a recent study, Hill and Smith (2023) discuss pre-pandemic approaches to blended learning at an institutional level, analyzing policy documents (e.g., strategic plans) and conducting interviews with expert practitioners. Their findings show that institutions tend to overestimate students’ and teachers ‘capacity to adapt to online learning tools just because it is assumed that living in a highly technologized and virtual-oriented society ensures one’s ability to be proficient in operating in online environments. This miscalculation can lead to failure in online learning engagement but can be overcome by the investment of institutions in change agendas based on strategy, structure and support (Hill and Smith, 2023).

### 2.3 Online learning and emergency remote teaching

Universities worldwide faced important challenges due to COVID 19 outbreak (Di Pietro et al., 2020). From problems attributed to shifting from face-to-face to online classes (both faculties and students had to deal with inadequate infrastructure needed for online learning; teachers had to redesign and rethink the methodologies in order to transform the online learning in a rich and effective learning experience for students, or in order to teach online practical and labs activities), to the impact on assessment and evaluation (new methods and techniques to fit to online learning mode), international students’ situation, or to the impact of this crisis on students’ mental health and wellbeing (Sahu, 2020) – the stakeholders had to make the best of a bad situation. In this note, Marinoni et al. (2020) found that universities have been confronted with a series of problems that influenced the feasibility and the quality of the distance learning they provided: technical infrastructure that is required to optimize distance learning both for the institutions and students; teachers’ lack of preparedness for effective change, the “learning by doing” approaches or attempting to imitate what would have been the face-to-face way of proceeding, yet using distance mode; the limitations of distance learning in some fields of study: clinical medicine, veterinary studies, and several disciplines depending on access to laboratories where hands-on practice cannot be replaced by distance teaching and learning.

There are differences between online learning and emergency remote teaching. While online learning, as previously described, provides content and tools that are *purposely designed* to improve the student’s learning experience in a virtual setting, emergency remote learning- as per its denomination – implies urgency, impetuous actions taken in an unforeseen situation. And while online learning is based on preparation and planned actions, emergency remote teaching requires fast yet provisional adaptation to transient learning conditions. While online learning assumes incremental progress evaluated in terms of learning outcomes (product evaluation) and educational procedural flow (process evaluation), remote emergency teaching tends to be more focused on needed resources, ongoing educational struggles, effectiveness of interactions and response to the newly (as in *unexpectedly*) created learning context (Hodges et al., 2020). Emergency remote learning (and teaching) is online learning but only by its infrastructural aspects. It lacks planning and designing for the long run, for educational objectives envisioning competency and personality development.

Online learning and emergency remote teaching might share the *remote* manner learning and teaching occur. But they differ in the freedom of choice and readiness of students and teachers. If online learning environments give, by design, teachers and students the comfort to choose the methods and means for teaching and own pace for learning, the emergency remote setting compels users to use online platforms regardless of teachers’ and students’ technical and technological capabilities (Sason et al., 2022). Even the “remote” word in *emergency remote teaching* suggests an instruction that is “removed” from its typical format and is adapted to an emergency (Hodges et al., 2021). This implies that *emergency remote teaching* may use not only online means for sustaining learning, but also other communication technology solutions for bridging physical distance between educators and students, such as mobile devices, local public television and other means of connecting (Moore and Hodges, 2023). Subsequently, this means that emergency remote teaching could often lack proper instructional design, due to the need of rapid adaptation to an unforeseen situation.

During the pandemic, universities that were not ready (infrastructure-ready, curricula-ready) for online learning faced challenges that translated into higher pressure on staff and students. On one hand, that is because during routine online learning support is more readily available than in case of emergency remote learning, as the courses have fewer students and teachers (Sason et al., 2022). On the other hand, the time available to prepare and manage learning situations in an emergency context is very limited and often insufficient, compared to the online learning systems that had the benefit of developing gradually.

The pandemic context was, in far too many cases, not an online learning approach, but a remote emergency teaching situation, where educational institutions were suddenly forced to move their business online, without precedent, without preparation, without benchmarking opportunities to evaluate and regulate online teaching performance and online learning satisfaction. That put arguably immense pressure on students and teachers to do things differently, but with the same desired results. In this context, learning itself has the potential of becoming a source of pressure, frustration and discomfort, the rapid transition from in person to online learning becoming a significant stressor (Reyes-Portillo et al., 2022). As Hodges et al. (2020) conclude, although educational challenges for all parties involved (students, staff, faculty) had been unlike anything in our lifetime, institutions will have the opportunity to assess the efficacy with which they were able to manage maintaining the continuity of instruction.

### 2.4 Academic stress

Given the pressured and challenges that students face during online learning, it is not unreasonable to think that they might experience academic stress as a result, with negative consequences on students’ wellbeing and their learning engagement.

Drawing from Lazarus and Folkman (1984) theory, we built upon the understanding that psychological stress can be seen as a response given by the person to a perceived unbalanced relationship between environmental requirements and the person’s resources to cope with those requirements. Thus, the response stems from external factors (seen as conditions or requirements) meeting one’s internal resources available for adequate response. A negative response in terms of inadequacy may result in a psychological reaction that can be referred to as stress. On this subject, Crosswell and Lockwood (2020) define stress as a cognitive, emotional and biological reaction to stressors. Stressors can be seen as discrete events having the potential to alter or disrupt typical psychological functioning (Crosswell and Lockwood, 2020). Referring to the academic context, Lazarevic and Bentz (2020) define stress as “the level of subjective perception of mental and emotional tension experienced by students while participating in the educational process.” But stressors may arise from various environmental contexts (academic and non-academic) and be conditioned by various personal configurations (different personality traits and experiential variables). Segerstrom and O’Connor (2012) talk about different approaches to the construct of stress and coping, naming role changing or role transitioning as one of the most important stressors a person would have to face. The appraisal of one’s ability to find and dispatch resources determines the level of stress one experiences. As for the stress managing process, Lazarus and Folkman (1984) describe coping as an adaptative effort that one employs, at cognitive and behavioral levels, to respond to circumstances appraised as exceeding one’s resources.

In an academic context, factors that are commonly indicated as stressors (triggering the negative response) have a complex *texture*, a composite nature. Academic stressors can include the students’ perception of the extensive knowledge base that is required and the perception of an inadequate amount of time to develop it (Misra and McKean, 2000). In developing their instrument, the Perception of Academic Stress Scale, Bedewy and Gabriel (2015) identify four factors related to academic stress among university students: (1) pressure to perform; (2) perceptions of workload; (3) academic self-perceptions; (4) time restraints. Therefore, from family’s expectations, perceived peer pressure and teachers’ requirements regarding academic performance (pressure to perform), to perceived excessive workload, limited time resources and academic self-confidence (a person’s expectations towards one’s own ability to properly respond to academic requirements, based on prior relevant experience), all were proven to be the main commonly identified sources of stress by students enrolled in university level programs, with no variance due to gender or across age groups (Bedewy and Gabriel, 2015).

As for the academic stressors associated with online learning *specifically* during the pandemic, a study conducted at King Saud University that examined the learning experience of 646 male and 438 female students, found six themes of concerns that were related to: *exams*, *learning assignments*, *lecture time*, *home and academic settings* for online learning, *the use of online platforms*, and students’ *uncertainty* about the fairness of online exams and assignments, about their ability to comprehend online lectures (Moawad, 2020). Among all respondents, the unease related to online learning tasks and examinations was reported as being the most prevalent of all identified stressors, results indicating that the uncertainty they feel regarding exams, the end of the semester and their assignments was the highest stressor affecting students (Moawad, 2020).

Online learning can be associated with lower levels of academic stress when explained by higher availability, flexibility and the possibility for customized learning solutions (Nath and Yadav, 2023) but can also add to the academic pressure especially in an emergency-type, rushed without alternative, context as was the pandemic one (Samudra and Matulessy, 2021). Online learning is in a mediated relationship with academic stress, with variables such as student’s perception of online learning, the readiness for online learning or the level of academic engagement acting as predictors. In that respect, online learning can become more stressful for students that have lower levels of readiness for online learning (Riaz et al., 2021), for students that express lower levels of academic engagement (Schaufeli et al., 2002) or for students that negatively evaluate their access to devices and technologies associated with online learning (Rosli et al., 2023). Online learning comes with specific challenges – the need for a higher level of self-management in learning, the scarcity of social interaction and unreliable internet access (Hermanto and Srimulyani, 2021), engagement for external isolated students that have poor access to online infrastructure (Gillett-Swan, 2017). Nonetheless, done right (learner oriented, teacher supportive), online learning presents diverse opportunities, such as accessibility, flexibility, interactivity and collaboration (Liang and Chen, 2012), that can lead to a more comfortable, stressless educational experience for users, both teachers and students. Therefore, online learning can have both positive and negative effects on students ‘outcomes (Lazarevic and Bentz, 2020).

### 2.5 Stress and wellbeing

In education, stress is relevant as it impacts not only students’ ability to perform academically, but also their mental health and well-being (Beiter et al., 2015). Wellbeing can be described as an ensemble of feelings, cognitions and strategies associated with positive functioning that contribute to physical and mental health (Kubzansky et al., 2023). Although the relationship between stress and wellbeing appears to be heavily mediated by multiple variables such as individual’s adaptive capacity, social support, cognitive abilities, knowledge, anticipatory socialization or preparation for events, the individual’s time perspectives, attitudes to self, and personality factors (Chalmers, 1982; Krause and Stryker, 1984; Segerstrom and O’Connor, 2012), there is sufficient evidence to suggest that stress is linked to wellbeing in a sense that high levels of stress are associated with impaired wellbeing (Bliese et al., 2017). Furthermore, recent research shows how appraisal of life events (positive or negative) can predict wellbeing (Chilver et al., 2023), specifically perceived stress was identified as a strong predictor of negative triad factors of psychological wellbeing (Klainin-Yobas et al., 2021). Concerning academic stress, recent studies have explored the impact of online learning during the COVID-19 pandemic on students’ academic stress and well-being indicating that academic stress is a significant negative predictor of well-being (Syed, 2021). More specifically, there is a significant relationship between academic stress and mental well-being among college students, particularly within online learning environments. Academic stressors, including heightened expectations, increased workload, and self-perception challenges, have been linked to diminished mental health outcomes, such as elevated anxiety and depression levels. For instance, a study found that students experiencing higher levels of stress during online learning reported poorer mental health outcomes, including increased anxiety and depression (Nuryana et al., 2023). Similarly, research has demonstrated that students reporting higher academic stress levels experience diminished mental well-being, regardless of demographic factors such as gender, race/ethnicity, or year of study (Barbayannis et al., 2022). Starting from these studies showing that academic stress as a predictor of mental health in university students, we chose to use the College Students Subjective Wellbeing Questionnaire (Renshaw, 2018) to help validate our instrument for perceived sources of stress in online learning. The selection of the College Student Subjective Wellbeing Questionnaire (CSSWQ) was informed by its validation as a tool for assessing college students’ well-being. Previous research has demonstrated significant correlations between academic stress and psychological well-being among college students. For instance, studies have found that higher levels of academic stress are associated with lower mental well-being (Córdova Olivera et al., 2023). While these studies did not specifically utilize the CSSWQ, the established relationship between academic stress and well-being supports the relevance of the CSSWQ in this context.

### 2.6 Stress and learning engagement

Learning engagement is a proactive decision that embodies feelings, thoughts, intentions and behaviors that a person manifests towards learning, as an activity. According to Schaufeli et al. (2002) engagement can be defined as a positive, fulfilling, work-related state of mind, characterized by vigor, dedication and absorption. Also, high levels of engagement have been proven to be associated with low levels of psychological distress (Schaufeli et al., 2002). In a study published in 2022, Stan et al. hypothesized a mediated predictive relationship between perceived stress and academic engagement in university students, in the sense that sources of stress negatively predict learning engagement The mediation model showed significant direct and indirect effects of sources of stress on learning engagement (Stan et al., 2022)., we used the UWES Learning Engagement Scale (Schaufeli et al., 2006) to help validate our instrument regarding students’ stress experienced in online learning. Extensive research has demonstrated a negative correlation between academic stress and student engagement. For instance, Agarwal et al. (2019) found that increased academic stress is associated with decreased engagement among medical students. Similarly, studies utilizing the Utrecht Work Engagement Scale (UWES) have shown that higher stress levels correspond to lower engagement scores. Given these findings, we employed the UWES Learning Engagement Scale (Schaufeli et al., 2006) to validate our instrument measuring students’ stress in online learning environments.

## 3 Aims

This study’s main objective was to develop and test the psychometric properties of the Sources of Stress in Online Learning Scale (SSOLS). Testing consisted in validation efforts aimed at establishing construct validity (through Exploratory Factor Analysis and Confirmatory factor analysis) and predictive validity of the instrument.

## 4 Method

### 4.1 Participants

The participants were 529 university students (*M* age = 23.76), attending various study domains (social sciences: 66.5%, engineering: 22.5%, medicine: 7.8%, economics: 3.2%) from several universities in Romania, and different educational levels: first year students (52.4%), second (27%), third and fourth year of study (20.6%). As for gender distribution, 87% of participants were female students. Most of the participants, 81%, were students at Transilvania University of Brașov and the University of Pitești, with 19% coming from other Romanian universities, such as West University of Timișoara or Ovidius University of Constanța. Although the sample was not randomized, the characteristics of the two main universities in the study are representative of comprehensive universities, with faculties covering all fundamental domains: engineering sciences, exact sciences, social sciences, arts and humanities, medicine, physical education, and sports. In addition, being situated in the center of the country, the variety of the student population is high, with students coming from nearly all regions, both urban and rural, and from high schools with different backgrounds and levels of achievement. Therefore, the sample could be considered relevant for the Romanian student population.

### 4.2 Measures

In developing the instrument, the authors reviewed relevant literature on challenges of online learning, observed and inquired their students about their experiences and the difficulties they were facing in the new circumstances of online learning, to identify the main stressors associated with online learning.

The SSOLS was administered online. Participants were invited to respond to the 32 items of the instrument using a 6 points Likert scale, conveying their own experience with online learning. Our study followed the principles of the *Declaration of Helsinki* regarding ethical aspects on human subjects. We did not collect any data that could lead to the identification of the participants. The participants gave their informed consent, while the participation at the study was specifically stated as being voluntary and no incentives were offered.

In the process and for the purpose of validating the new scale, two additional instruments were used: College Student Subjective Wellbeing Questionnaire (CSWQ, Renshaw, 2018) and UWES Learning Engagement Scale (Schaufeli et al., 2006).

The College Student Subjective Wellbeing Questionnaire (CSSWQ) is a self-report measure designed to assess domain-specific well-being in college students’ academic lives (Renshaw, 2018) consisting of 16 items assessed using a Likert scale that ranges between 1 – to a small extent and 5 – to a very large extent and measuring four dimensions, with four items included in each dimension: academic efficacy (the items assess students’ confidence in their ability to perform and succeed in academic tasks, *α* = 0.84), academic satisfaction (the four items assess students’ confidence in their ability to perform and succeed in academic tasks, α = 0.85), academic connectedness (the items evaluate the sense of belonging and quality of relationships within the college community, α = 0.74), and academic gratitude (the …items measure the extent of positive emotions and appreciation related to the college experience, α = 0.80). Cronbach’s Alpha for the entire scale was 0.91.

Learning engagement was measured with the UWES Learning Engagement Scale (Schaufeli et al., 2006). UWES assesses students’ academic engagement, and it is composed of 9 items that assess: vigor (3 items, α = 0.89), absorption (3 items, α = 0.74) and dedication (3 items, α = 0.80), each item is assessed using a Likert scale that ranges between 1 – to a small extent and 5 – to a very large extent. Cronbach’s Alpha for the total scale is 0.92.

### 4.3 Data analysis

To test the construct validity of the scale, the convenience sample was randomly split into exploratory (*N* = 271) and confirmatory (*N* = 258) samples, the two halves not differing on gender (χ^2^ = 0.12, *p* = 0.72), educational level (χ^2^ = 2.48, *p* = 0.47), study domain (χ^2^ = 6.52, *p* = 0.16), all the χ2 tests being nonsignificant. Exploratory and confirmatory factor analysis were used to investigate the construct validity of the scale and linear regression to estimate the predictive validity. Exploratory factor analysis was computed using JASP 16.4.0 Promax with Kaiser Normalization being computed and Confirmatory factor analysis was computed also with JASP.

## 5 Results

### 5.1 Construct validity

The first version of the SSOLS included 32 items. An initial Exploratory Factor Analysis was computed. Based on the eigenvalues, Kaiser’s rule, and the scree plot, a solution with seven factors was assumed. The Kaiser–Meyer–Olkin (KMO = 0.912) and Bartlett’s test of sphericity (χ2 = 4591.6, *p* < 0.001) indicated that the data were suitable for exploratory factor analysis. The seven factors covered 64.32 of the total variance. The factors were labelled as follows: F1 Inadequacy of teaching methods and teaching styles, F2 Lack of social support, F3 Technical difficulties, F4 Role conflict, F5 Time constraints, F6 Diversity of techniques, F7 Inflexibility. Four items were eliminated because of their saturations in more than two factors, their structure being ambiguous. All the items had loadings higher than 0.47. The low reliability of the seventh factor also suggests the possibility to eliminate the two items (Table 1). For each subscale, item-level analyses were conducted (Table 1). For F1, Inadequacy of teaching methods and teaching styles, all item-total correlations were higher than 0.55, for F2 Lack of social support, 0.47, for F3 Technical difficulties, 0.43, for F4 Role conflict, 0.37, for F5 Time constraints, 0.63, for F6 Diversity of techniques, 0.58 and for F7 Inflexibility, 0.31.

**Table 1:** Exploratory factor analysis for SSOLS items.

Items	*M*	*SD^2^*	*r*	Loadings						
				F1	F2	F3	F4	F5	F6	F7
A24. The teaching methods are not adapted to the online mode.	3.00	2.00	0.79	0.78						
A19. The teachers are more focused on the activity and the assignments than on the students.	3.38	2.00	0.78	0.77						
A13. The teachers have different perspectives on online assignments.	2.77	1.84	0.76	0.76						
A25. The teachers do not provide us with diverse digital learning resources.	2.55	1.66	0.73	0.76						
A18. The teachers’ emotional involvement is low.	2.72	1.85	0.75	0.75						
A17. The teachers do not give immediate feedback to our homework and assignments.	2.67	1.93	0.62	0.74						
A16. It is difficult to get clarifications from teachers in real time.	2.94	1.87	0.73	0.71						
A23. The way the subject/ content is organized is not adapted to online learning.	3.28	1.98	0.76	0.70	0.37					
A5. Not all my teachers have high skills in using the technology and digital teaching-learning.	2.96	2.09	0.63	0.69						
A12. Teachers’ expectations in online learning are unclear to me.	3.48	1.87	0.71	0.63	0.30					
A14. It is difficult to benefit from online consultations from my teachers.	2.74	1.79	0.64	0.61						
A8. The university’s e-learning platform works poorly.	3.28	1.96	0.55	0.61						
A26. There is a large and various amount of tasks that we need to solve for each subject.				0.59	0.41		0.33			
A28. The deadlines for posting the solved tasks on the e-learning platform are quite “tight.”				0.48	0.32		0.46			
A21. I feel the lack of encouragement and support from teachers in facilitating learning.	3.26	2.33	0.684	0.37	0.75					
A20. I feel the lack of interactions and debates with colleagues in solving academic tasks.	3.5	2.21	0.708		0.74					
A22. Online learning gives me the feeling that I am rather isolated and not belonging to a learning community.	3.25	2.35	0.685		0.73					
A30. I have doubts about my ability to cope with online assessment.	3.58	2.05	0.476		0.47			0.373	−0.321	
A27. I need to be constantly connected to keep up with the information. Topics and tasks posted for each subject.				0.30	0.46		0.35			0.30
A29. I have uncertainties about the way the final evaluation will be conducted.				0.41	0.42				−0.36	
A7. I do not possess a high-performance device to support the online learning.	2	1.63	0.659			0.84				
A6. I have limited/ difficult internet access.	2.13	1.69	0.583			0.79				
A1. I have low technical skills in using digital learning.	1.99	1.34	0.434			0.57				0.47
A32. My work activities on learning platforms overlaps with my professional responsibilities.	2.74	2.38	0.372				0.68		0.43	
A31. My work activities on learning platforms overlaps with my family responsibilities.	3.46	1.88	0.372				0.63	0.30		
A11. I cannot benefit from a recording of the conferences I missed.				0.35			0.45		−0.31	
A4. I do not have a rigorous learning program.	3.15	1.80	0.631					0.81		
A3. I tend to postpone academic assignments.	2.86	2.06	0.631					0.80		
A9. The teachers use a too wide variety of learning platforms.	2.35	1.67	0.580						0.71	
A15. The teachers use too many ways to convey information.	2.49	1.69	0.580	0.32					0.63	
A2. I have to use tools specific to digital learning, during the activities on the e-learning platform (microphone. Video, chat, sharing materials, etc.).	3.18	2.28	0.310							0.80
A10. I have to be present on the platform at a certain time, which I do not decide.	3.33	2.26	0.310				0.39			0.59
Variance explained				24.19	10.64	6.37	6.36	6.21	5.48	5.04
Eigenvalues				11.09	2.56	1.80	1.48	1.33	1.19	1.11
Number of items retained after EFA				13	4	3	2	2	2	2
Cronbach’s Alpha				0.92	0.81	0.73	0.60	0.77	0.75	0.50

Extraction Method: Principal Component Analysis. Rotation Method: Varimax with Kaiser Normalization. r = item-total correlation. F1 = Inadequacy of teaching methods and teaching styles, F2 = Lack of social support, F3 = Technical difficulties, F4 = Role conflict, F5 = Time constraints, F6 = Diversity of techniques, F7 = Inflexibility.

Based on the EFA results, we confirmed the structure of the questionnaire through CFA. Given the low reliability of the seventh factor, we tested a Confirmatory factor analysis with six factors (Table 2). The factor structure showed good fit for several indices after eliminating item 26 and after adding covariances between errors as indicated by the modification indices (Root Mean Square Error of Approximation, RMSEA = 0.05; Comparative Fit Index, CFI = 0.93, Tucker–Lewis Index, TLI = 0.92, CMIN = 1.70).

**Table 2:** Confirmatory factor analysis for SSOLS items.

Items	*B*	*St. er.*	*β*	*z*	*p*
F1 Inadequacy of teaching methods and teaching styles					
A24. The teaching methods are not adapted to the online mode.	1		0.71	0.93	<0.001
A19. The teachers are more focused on the activity and the assignments than on the students.	1.05	0.09	0.73	11.31	<0.001
A13. The teachers have different perspectives on online assignments.	1.11	0.09	0.76	11.78	<0.001
A25. The teachers do not provide us with diverse digital learning resources.	0.90	0.07	0.66	12.9	<0.001
A18. The teachers’ emotional involvement is low.	0.94	0.09	0.64	9.81	<0.001
A17. The teachers do not give immediate feedback to our homework and assignments.	0.84	0.09	0.56	8.67	<0.001
A16. It is difficult to get clarifications from teachers in real time.	0.87	0.09	0.59	9.05	<0.001
A23. The way the subject/ content is organized is not adapted to online learning.	0.88	0.06	0.65	14.03	<0.001
A5. Not all my teachers have high skills in using the technology and digital teaching-learning.	0.94	0.10	0.60	9.30	<0.001
A12. Teachers’ expectations in online learning are unclear to me.	1.14	0.09	0.80	12.4	<0.001
A14. It is difficult to benefit from online consultations from my teachers.	0.94	0.09	0.64	9.89	<0.001
A8. The university’s e-learning platform works poorly.	0.84	0.09	0.61	9.38	<0.001
F2 Lack of social support					
A21. I feel the lack of encouragement and support from teachers in facilitating learning.	1		0.80	1.13	
A20. I feel the lack of interactions and debates with colleagues in solving academic tasks.	0.89	0.07	0.72	11.69	<0.001
A22. Online learning gives me the feeling that I am rather isolated and not belonging to a learning community.	0.92	0.08	0.69	11.11	<0.001
A30. I have doubts about my ability to cope with online assessment.	0.82	0.07	0.70	11.34	<0.001
F3 Technical difficulties					
A7. I do not possess a high-performance device to support the online learning.	1		0.60	0.73	
A6. I have limited/ difficult internet access.	1.08	0.12	0.62	8.75	<0.001
A1. I have low technical skills in using digital learning.	1.16	0.17	0.71	6.81	<0.001
F4 Role conflict					
A32. My work activities on learning platforms overlaps with my professional responsibilities.	1		0.57	0.92	<0.001
A31. My work activities on learning platforms overlaps with my family responsibilities.	1.29	0.21	0.86	6.10	<0.001
F5 Time constraints/lack of time management skills					
A4. I do not have a rigorous learning program.	1		0.90	1.16	
A3. I tend to postpone academic assignments.	0.83	0.10	0.69	7.89	<0.001
F6 Diversity of techniques					
A9. The teachers use a too wide variety of learning platforms.	1		0.81	1.04	
A15. The teachers use too many ways to convey information.	0.93	0.12	0.76	7.72	<0.001

The item 26 with low loading was removed because it did not meaningfully contribute to the underlying construct. Similarly, the addition of error covariances was based on modification indices provided by the CFA results (Byrne, 2016; Kline, 2015). The CFA analysis revealed a better solution. In the final structure, all the items had loadings higher than 0.56 for all the seven factors, conform the exploratory factor solution. Three retained factors have only two items (F4, F5, and F6). While multi-item scales are generally preferred in psychological and educational research, we decided to retain the two-item factors based on both theoretical and empirical considerations. Inter-item correlations and reliability estimates indicated acceptable internal consistency. Furthermore, these two-item factors represent well-defined, narrow constructs with strong conceptual coherence, reflecting specific dimensions of students’ stress in online learning. Research supports the use of two-item scales when items are highly homogenous and directly aligned with the measured construct (Eisinga et al., 2013). However, we acknowledge that two-item factors may pose limitations regarding factorial stability and reliability, particularly across different populations or over time. Therefore, we recommend that future development of the SSOLS scale should consider expanding these factors by including additional items, in order to enhance the psychometric robustness and generalizability of the instrument.

The Pearson correlations between the six factors were statically significant (*p* < 0.001), the highest correlation being obtained between F1 Inadequacy of teaching methods and teaching styles and F2 Lack of social support, F2 and F5 Time constraints, and the lowest correlation between F5 Time constraints and F6 Diversity of techniques (Table 3).

**Table 3:** Pearson correlation coefficients between SSOLS dimensions, academic wellbeing and learning engagement.

Dimensions	1	2	3	4	5	6	7	8	9	10	11	12	13
1.SSOLS inadequacy of teaching	1												
2.SSOLS lack of support	0.60^***^	1											
3.SSOLS technical difficulties	0.29^***^	0.36^***^	1										
4.SSOLS role conflict	0.27^***^	0.23^***^	0.35^***^	1									
5.SSOLS time constraints	0.40^***^	0.42^***^	0.20^***^	0.18^***^	1								
6.SSOLS diversity	0.32^***^	0.20^***^	0.34^***^	0.35^***^	0.16^***^	1							
7.CSWQ academic efficacy	−0.22^***^	−0.18^***^	−0.05	−0.08^*^	−0.42^***^	−0.03	1						
8.CSWQ school connectedness	−0.36^***^	−0.23^***^	−0.18^***^	−0.16^***^	−0.24^***^	−0.09^*^	0.55^***^	1					
9.CSWQ college gratitude	−0.26^***^	−0.10^*^	−0.10^*^	−0.20^***^	−0.14^***^	−0.17^***^	0.40^***^	0.64^***^	1				
10.CSWQ academic well-being	−0.34^***^	−0.22^***^	−0.14^***^	−0.18^***^	−0.34^***^	−0.11^*^	0.81^***^	0.86^***^	0.75^***^	1			
11.UWES vigor	−0.48^***^	−0.44^***^	−0.11^**^	−0.17^***^	−0.43^***^	−0.05	0.46^***^	0.46^***^	0.36^***^	0.52^***^	1		
12.UWES dedication	−0.46^***^	−0.42^***^	−0.17^***^	−0.20^***^	−0.38^***^	−0.10^*^	0.47^***^	0.50^***^	0.46^***^	0.57^***^	0.81^***^	1	
13.UWES absorption	−0.28^***^	−0.27^***^	−0.14^***^	−0.18^***^	−0.33^***^	−0.11^*^	0.48^***^	0.44^***^	0.36^***^	0.51^***^	0.66^***^	0.77^***^	1
14.UWES learning engagement	−0.45^***^	−0.42^***^	−0.16^***^	−0.20^***^	−0.42^***^	−0.09^*^	0.51^***^	0.51^***^	0.43^***^	0.59^***^	0.91^***^	0.945^*^	0.88^***^

*N* = 529, *p < 0.05, **p < 0.01, ***p < 0.001. SSOLS = Sources of Stress in Online Learning Scale; CSWQ = The College Student Subjective Wellbeing Questionnaire; UWES = The UWES Learning Engagement Scale.

### 5.2 Convergent and concurrent validity

The associations between SSOLS dimensions and academic wellbeing were low to moderate but statically significant, Inadequacy of teaching methods and Time constraints showing the strongest associations (Table 3). The results demonstrate a clear pattern of negative associations between various sources of academic stress and students’ academic well-being. Across all dimensions of stress, there is a consistent tendency for higher levels of stress to be linked with lower levels of well-being. Specifically, the perception of inadequate teaching appears to undermine students’ academic efficacy, school connectedness, college gratitude, and overall well-being. When students feel that instructional quality is insufficient, their sense of competence, connection to their institution, and appreciation for their academic experience tend to decrease. Similarly, a lack of support is associated with diminished academic well-being. Students who perceive limited support are less likely to feel connected to their academic environment and may struggle more with maintaining a positive outlook on their educational experience. Technical difficulties also show a negative relationship with well-being, although these associations appear to be weaker compared to other stressors. This suggests that, while technical challenges can be disruptive, they may not be as central to students’ overall well-being as other stress factors. Time constraints emerge as a particularly impactful stressor, with strong connections to reduced academic efficacy and overall well-being. The pressure to manage multiple academic responsibilities can erode students’ confidence in their abilities and contribute to a general decline in their academic well-being. Role conflict also shows a detrimental relationship with well-being, indicating that difficulties in balancing academic demands with other responsibilities can compromise students’ engagement and sense of satisfaction with their academic experience. Lastly, stress related to diversity, although present, appears to have a less pronounced effect on academic well-being. While feelings of dissonance related to diverse learning environments could impact well-being, their predictive values is comparatively weaker than that of other stressors.

The correlation coefficients were higher for learning engagement (Table 3). The correlations indicate a consistent negative relationship between academic stress and learning engagement. When students perceive inadequate teaching, lack of support, or encounter technical difficulties, their levels of vigor, dedication, absorption, and overall engagement tend to decrease. Time constraints and role conflict also emerge as significant factors, as students struggling to manage multiple responsibilities or facing tight deadlines often report lower engagement in their academic tasks. While diversity-related stress also shows a negative association with engagement, its impact appears less pronounced compared to other stressors.

To test the predictive validity of the SSOLS, a multiple linear regression was performed (Table 4), predicting the general score of academic wellbeing through the six factors of the scale. The predictive validity was weak, the six factors predicting 17% of the wellbeing variance, only factors 1 and 5 having a significant negative weight. Other variables such as personal coping strategies, social support, or resilience likely play important roles and should be integrated in future models.

**Table 4:** Regression analysis for the prediction of academic wellbeing (Model 1) and learning engagement (Model 2).

Model	Predictors	*B*	*Std. Error*	*β*	*t*	*p*
1	Constant	4.87	0.11		41.32	<0.001
	1.Inadequacy of teaching	−0.19	0.03	−0.26	−5.01	<0.001
	2.Lack of support	0.03	0.03	0.06	1.15	0.25
	3.Technical difficulties	−0.02	0.03	−0.02	−0.57	0.55
	4.Role conflict	−0.04	0.02	−0.08	−1.81	0.07
	5.Time constraints	−0.14	0.02	−0.24	−5.49	< 0.001
	6.Diversity	0.02	0.02	0.04	0.92	0.35
Dependent Variable: Academic wellbeing, *R* = 0.42. *R*^2^ = 0.17, *F*(6,528) = 18.61, *p* < 0.001						
2	Constant	4.49	0.14		31.89	< 0.001
	1.Inadequacy of teaching	−0.25	0.04	−0.27	−5.59	< 0.001
	2.Lack of support	−0.13	0.04	−0.16	−3.31	0.001
	3.Technical difficulties	0.02	0.04	0.03	0.71	0.47
	4.Role conflict	−0.06	0.03	−0.09	−2.20	0.02
	5.Time constraints	−0.18	0.03	−0.24	−5.88	< 0.001
	6.Diversity	0.07	0.03	0.09	2.17	0.03
Dependent Variable: Learning engagement, *R* = 0.54. *R*^2^ = 0.30, *F*(6,528) = 37.21, *p* < 0.001						

For the learning engagement, the prediction was higher, the six factors predicting 30% of the total engagement, with all the SSOLS dimensions except F3 being significant. The results showed that high values for five of the six dimensions predict low learning engagement, while high levels of diversity of techniques predicts a higher engagement.

## 6 Discussion and conclusion

As stated in previous sections, even though the major global health emergency is currently over, the difficulties in managing educational systems brought to light by the last three complicated years remain. Yet with challenges come opportunities. As the academic community has grown fonder of online education over the past decade, online learning has become part of the student’s learning repertoire. So, it is here to stay. That is why focusing on ways to improve the online academic process is mandatory. At the heart of it stands a strong evaluation process, which can determine educational needs, specific means and tools to properly address it. Students’ stress related to academic learning is one of the variables that can prove to be crucial for both better understanding and significantly improving the students’ learning experience, thus enhancing the quality of teaching and the quality of services educational institutions provide.

Although instruments for evaluating students’ stress in an academic environment exist (e.g., Bedewy and Gabriel, 2015), thus far we were unable to identify reliable and valid instruments for perceived sources of stress in online learning. The advantage of having a robust instrument to assess learning related stress in online contexts is that it provides stakeholders valuable and accurate insight into the “what can go wrong” factors relating to the process of online learning, from the beneficiary’s point of view. In addition, this insight can prove useful for designing educational contexts that reduce stress and improves academic engagement in students. It is important because stress is shown to be associated with low level engagement (Schaufeli et al., 2002) and low-level wellbeing (Bliese et al., 2017; Klainin-Yobas et al., 2021; Chilver et al., 2023), variables that are key factors in the academic success equation. An option to consider is integrating mental health support programs into the university curriculum. Recent studies have demonstrated that integrating mindfulness-based stress reduction techniques into the school curriculum, particularly in the aftermath of the COVID-19 pandemic, can significantly reduce student stress levels and improve overall mental well-being (Norwich et al., 2022).

The primary aim of this research was to develop and validate an instrument for measuring sources of stress in online learning, with the factorial structure of the scale serving as a central focus of the study.

The initial construction of the scale for sources of stress in online learning was based on the dimensions most frequently mentioned in the specialized literature in recent years, particularly following the COVID-19 pandemic, which significantly increased the prominence of online learning in students’ academic lives. From a statistical perspective, the use of both Exploratory Factor Analysis and Confirmatory Factor Analysis enabled us to identify the factorial structure of the SSOLS. The analysis revealed six distinct factors: Inadequacy of teaching methods and teaching styles, Lack of social support, Technical difficulties, Role conflict, Time constraints, and Diversity of techniques. Notably, the strongest correlation was observed between Inadequacy of teaching methods and Lack of social support, suggesting the critical role of instructional quality and social connections in shaping students’ stress experiences. In contrast, the weakest correlation was found between Time constraints and Diversity of techniques, indicating that the pressure of time constraints is relatively independent from the variety of techniques used in online learning. These findings provide a more nuanced understanding of the sources of stress in online learning environments and highlight potential areas for intervention.

For introducing an instrument with good psychometric properties, we have collected relevant data from more than 500 university students attending various academic programs, from Psychology, Educational Sciences, to Medicine, Economics and Engineering, and subsequently conducted a complex analysis aimed at ensuring that the scale had proper consistence and validity, and thus can be trusted. As for our results, the SSOLS performed well as it proved to be a reliable instrument measuring students’ perceived sources of stress associated with online learning. The development and validation of the Sources of Stress in Online Learning (SSOLS) scale aimed to create an instrument that accurately reflects the diverse factors contributing to stress in digital learning environments. The SSOL identifies key stress dimensions, including inadequacy of teaching methods and styles, lack of social support, time constraints, technical difficulties, role conflict, and diversity of techniques. By delineating these factors, the study contributes to the theoretical discourse on the psychological and educational dynamics of online learning.

Furthermore, the dimensions of SSOLS were shown to be valid predictors for academic engagement. Specifically, when the students reported high levels for most stressors associated with online learning, their engagement level had low values. That means that when stress is perceived as springing from those sources, the learning engagement tends to drop. As for the sixth dimension- Diversity - the association was positive, meaning that a high level of perceived diversity regarding techniques employed in teaching correlates with a high level of engagement. In short, high diversity in online learning drives high learning engagement. The SSOLS was successfully used in a study we recently published as it allowed us to predict learning engagement in university students (Stan et al., 2022). The good predictive value of the SSOLS’s stress factors for learning engagement is in accordance with Schaufeli et al. (2002) study that found a strong association between engagement and distress.

For validity purposes, we also tested the predictive value of the SSOLS dimensions for students perceived academic wellbeing level. In the first model, the stress sources factors proved to be weak in predicting wellbeing, the six factors predicting 17% of the wellbeing variance. We attribute this, in part, to the mediated nature of the relation between perceived stress and wellbeing (Chalmers, 1982; Krause and Stryker, 1984). However, in this regression analysis, factor 1 (*Inadequacy of teaching*) and factor 5 (*Time constraints*) have been found to have a significant negative weight in predicting wellbeing.

For both models, factor 3 (*Technical difficulties*) had the weakest association (amongst all five factors) with academic wellbeing and learning engagement. We assume the reasons for this result can be found in the factor composition and in the sample’s demographic features. Most of the respondents were emergent adults with a high level of digital proficiency, likely reducing the impact of technical barriers on their online learning experience. As the Technical Difficulties factor mainly captured access to high-performance devices and reliable internet, it may have been less relevant for a digitally fluent, university-aged population. We acknowledge this limitation and suggest that future research should validate the SSOLS in more diverse populations, including students with varying degrees of technological access and skills, to better understand the contextual relevance of technical stressors. Also, for both models, factor 1 (*Inadequacy of teaching*) and factor 5 (*Time constraints*) behaved as strong predictors for learning engagement and wellbeing. An explanation for this result can be attributed to the preponderant importance students place on teaching aspects (teacher’s skills, expectations, methods, communication availability, etc.) pertaining to the learning experience and time pressure the students experience in educational contexts, in accordance with findings from Bedewy and Gabriel (2015) and Moawad (2020).

Overall, the results highlight the detrimental impact of academic stress on learning engagement. Among the various stressors, time constraints, inadequacy of teaching, and lack of support stand out as the most influential, suggesting that these areas may be particularly important targets for interventions aimed at enhancing student engagement in online learning contexts. Several studies have highlighted the detrimental impact of academic stress on student engagement, particularly in online learning contexts. Factors such as time constraints, inadequate teaching, and lack of support have been identified as significant stressors that negatively affect student engagement (Stan et al., 2022). Similarly, the absence of emotional support from teachers has been linked to decreased learning engagement among college students. A recent study exploring this relationship found that teacher support plays a crucial role in fostering student engagement, especially in challenging learning environments (Guo et al., 2025). These findings suggest that addressing time constraints, enhancing teaching quality, and providing adequate support are essential strategies for mitigating academic stress and promoting better engagement in online learning settings.

The findings for the prediction of academic well-being suggest that academic stress plays a significant role in shaping students’ well-being, with certain stressors, such as time constraints, inadequate teaching, and lack of support, standing out as particularly influential. Research indicates that academic stress significantly influences students’ well-being, with stressors such as time constraints, inadequate teaching, and lack of support being particularly impactful. For instance, high levels of stress can impair cognitive functions like attention and memory, leading to difficulties in concentration and information retention, which adversely affect academic performance and overall well-being (Córdova Olivera et al., 2023). Addressing these stressors through targeted interventions can foster a more supportive and engaging learning environment, especially in online settings. Implementing adaptive coping strategies, including social and emotional support, has been found to improve students’ mental well-being. Stress-reduction peer support groups and workshops on campus could be beneficial in reducing stress and enhancing self-efficacy among students (Barbayannis et al., 2022). By focusing on these areas, educational institutions can develop strategies that mitigate academic stress and promote better well-being among students.

Limitations for this study arise from sampling respondents and from the self-reporting, thus intrinsically subjective nature of the data collection procedure. First-year students were strongly represented (52.4%), while third- and fourth-year students were slightly underrepresented (20.7%). Freshmen may experience heightened stress due to the challenges associated with transitioning to university life and adapting to online learning, while juniors and seniors might face increased academic demands and concerns related to graduation or future career plans. We acknowledge this sample imbalance as a limitation that may affect the generalizability of our findings. Future research should consider academic level as a potential mediating factor when examining stress in online learning environments (Hadwin et al., 2022).

Additionally, regarding gender distribution, female students made up 87% of the total respondents, with male students being underrepresented. This gender imbalance may introduce biases in perceived stress and engagement levels, as prior research suggests that gender influences academic stress experiences (Misra and McKean, 2000). Consequently, the findings may not fully generalize to male students or to more gender-diverse populations. Future studies should aim for a more balanced gender distribution to improve external validity and provide a more nuanced understanding of gender-specific stress factors in online education.

In addition, the absence of exclusion criteria and the lack of randomization in the sampling process chosen to achieve a high variability of student participants, may affect the external validity of the study and limit the generalizability of the results. Furthermore, the non-randomized sampling approach may introduce selection bias, as certain types of students could have been more likely to participate. Improvements can be brought by purposely ensuring and enhancing the diversity of the respondent students’ demographic, social, educational, professional backgrounds, students’ ethnicity, and nationality. Given the uneven group sizes, multigroup analyses were not computed in this study, the characteristics of the sample could have led to estimation issues, such as non-convergence or inflated Type I errors, as documented in the literature (Byrne, 2016; Kline, 2015). Therefore, future research with larger and more balanced samples should further explore these group differences.

Exclusive reliance on self-report measures as a limitation of the present study, which may introduce biases such as social desirability or subjective misreporting. To strengthen future research, we recommend incorporating multiple data sources, such as behavioral measures, academic performance records, and clinical assessments, alongside self-reports. Triangulating findings across these diverse methods would provide a more robust and comprehensive understanding of the stress factors associated with online learning. This approach will provide a more robust and comprehensive understanding of the stress factors associated with online learning.

Another possible limitation refers to the factor structure of the scale. Although the retained factors led to good fit indices and consistence with the theoretical considerations guiding the development of this scale, some factors had only two items which can lead to instability, which is an important limitation. To address this, we are considering revising the scale by adding more items to these factors in future studies. Our aim is to include four to five items per factor to ensure more comprehensive coverage of the constructs and better scale reliability.

In this exploratory study, we focused our attention on the stress factors associated only with online learning. To gain more comprehensive insight regarding the level and nature of stress experienced by students studying online, a study comparing the differences between the perceived stress of online learners and that of the learners from conventional face-to-face instruction should be conducted.

Also, for future development, the instrument that has been developed can be used in studies with correlational or predictive research designs, aiming at discovering the most effective strategies for optimizing the online learning experience for students and teachers while making the most judicious use of the available institutional resources. Understanding the sources of stress among university students involved in online learning is an important step in dealing with this problem and developing pedagogical designs of online courses which can foster student’s productive participation.

The findings of this research have direct implications for educational systems and policymakers. By accurately assessing the primary sources of academic stress, institutions can implement more targeted interventions to support students’ mental health and academic performance. For instance, improving teacher training programs to address pedagogical inadequacies, fostering stronger peer and instructor support networks, and developing more flexible and adaptable curricula to mitigate time constraints can significantly enhance the online learning experience. Ultimately, the SSOLS scale not only provides researchers with a valuable tool for further studies but also equips educators with actionable insights to create more supportive, engaging, and effective learning environments.

The significance of this study lies also in its contribution to both theoretical understanding and practical applications regarding academic stress in online learning environments. As the online trend in education continues to grow and expand, being able to identify possible impediments and challenges that can be addressed by teachers and students together is paramount for the process’s effectiveness. It thus becomes imperative to have means of identifying and measuring levels of stressors -as burdening sources of dissatisfaction with the learning experience - and to better manage the lucrative opportunity of predicting the influence they may have on academic engagement and consequently on academic performance.

## Data Availability

The original contributions presented in the study are included in the article/supplementary material, further inquiries can be directed to the corresponding author/s.
